# Tissue Factor/Factor FVII Complex Inhibitors in Cardiovascular Disease. Are Things Going Well?

**DOI:** 10.2174/157340310793566190

**Published:** 2010-11

**Authors:** Gianluca Petrillo, Plinio Cirillo, Greta-Luana D’Ascoli, Fabio Maresca, Francesca Ziviello, Massimo Chiariello

**Affiliations:** Department of Internal Medicine, Cardiovascular and Immunological Sciences (Division of Cardiology) University of Naples "Federico II", Italy

**Keywords:** Blood coagulation, cardiovascular disease, factor VIIa, tissue factor.

## Abstract

Blood coagulation is a complex biological mechanism aimed to avoid bleeding in which a highly regulated and coordinated interplay of specific proteins and cellular components respond quickly to a vascular injury. However, when this mechanisms occurs in the coronary circulation, it has not a “protective” effect, but rather, it plays a pivotal role in determining acute coronary syndromes. Coagulation recognizes Tissue Factor (TF), the main physiological initiator of the extrinsic coagulation pathway, as its starter.

Since TF:VIIa complex is the critical point of the blood coagulation cascade, it is a pharmacological attractive issue for the development of agents with anti thrombotic properties that can exert their activity by inhibiting complex formation and/or its catalytic activity. In fact, it is intuitive that an antithrombotic agent able to inhibit this initial step of the coagulation pathway has several theoretical, extremely important, advantages if compared with drugs active downstream the coagulation pathway, such as FXa or thrombin. The present report gives a brief overview of TF pathophysiology, highlighting the most recent advances in the field of inhibitors of the complex TF/VIIa potentially useful in cardiovascular disease.

## INTRODUCTION

Blood coagulation is a complex biological mechanism aimed to avoid bleeding in which a highly regulated and coordinated interplay of specific proteins and cellular components respond quickly to a vascular injury. However, when this mechanism occurs in the coronary circulation, it has not a “protective” effect, but rather, it plays a pivotal role in determining acute coronary syndromes [1].

Coagulation recognizes Tissue Factor (TF), the main physiological initiator of the extrinsic coagulation pathway, as its starter. Indeed, several experimental and clinical studies indicate that TF plays a pivotal role in the pathophysiology of acute coronary syndromes: it triggers the formation of intracoronary thrombi following endothelial injury [2-5]. TF is an integral transmembrane protein expressed on the surface of several cell types located in subendothelial structures throughout the vasculature, and it is normally not in contact with circulating blood, where other coagulation factors are present in their inactivated forms. In this respect, cells normally not exposed to the flowing blood, such as smooth muscle cells, constitutively express TF on their surface [6, 7], while cells exposed to the blood stream, such as endothelial cells, express TF on their membrane only when activated after exposure to specific stimuli, such as LPS, certain cytokines [8], and oxygen free radicals [9].

Since TF/VIIa complex formation represents the critical point of the blood coagulation cascade, it is an attractive tool for the development of agents with anti thrombotic properties, which could inhibit complex formation and/or its catalytic activity. Indeed, it is intuitive that an antithrombotic agent able to inhibit this initial phase of the coagulation pathway has several theoretical, extremely important, advantages if compared with drugs active downstream the coagulation pathway, such as FXa or thrombin, since this pathway is specifically blocked right from the beginning.

The present report gives a brief overview of TF pathophysiology, highlighting the recent advances in the field of inhibitors of the TF/VIIa complex useful in cardiovascular disease treatment.

### Tissue Factor Physiology and Extrinsic Coagulation Pathway

**Fig. (1):**
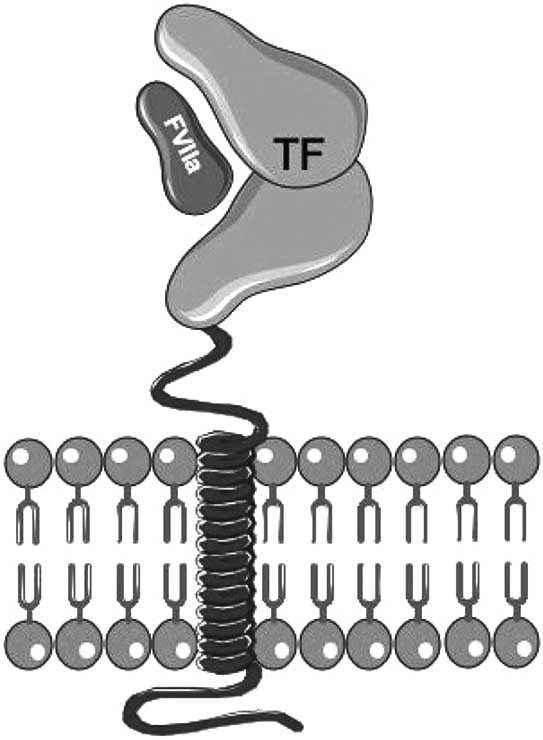
Schematic representation of Tissue Factor. This glycoprotein is a type I integral membrane protein. The extracellular part of TF binds FVII with very high affinity and specificity. Once bound to TF, FVII is rapidly converted to its activated form (FVIIa) *via* limited proteolysis. Membrane anchoring of TF has been demonstrated to be essential to support full proteolytic activity by FVIIa.

**Fig. (2):**
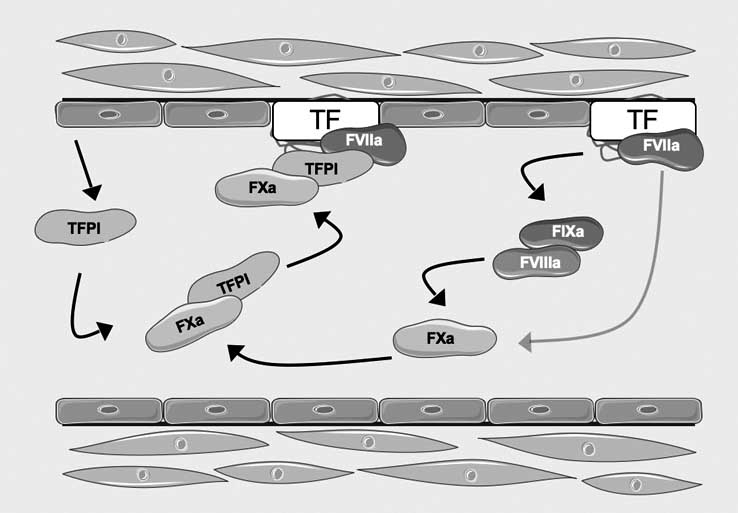
Schematic representation of Extrinsic coagulation pathway: FVII bound to TF is rapidly converted to its activated form (FVIIa). FVIIa binds FX, that is converted in its activated form (FXa). Many coagulation proteases such as factors VIIa, IXa, amplify this activation process. TFPI modulates the TF/FVIIa complex activity. In presence of FXa, it forms a complex which, in turn, binds with high affinity to TF/VIIa thus causing the formation of a fully inhibited tetra-molecular complex TF/VlIa/TFPI/Xa.

Tissue Factor (TF), also known as thromboplastin or CD142, is a glycosylated transmembrane protein consisting of a single polypeptide chain (MW: 45,000) [10]. This glycoprotein is a type I integral membrane protein, and is a member of the class 2 cytokine receptor superfamily [11]. The extracellular part of TF is made up of two fibronectin type III domains, and membrane anchoring of TF has been demonstrated to be essential to support full proteolytic activity by FVIIa [12] (Fig. **1**). It is intuitive that TF is normally not exposed to circulating blood to avoid its improper interaction with other circulating coagulation factors. Vice versa, vascular injury, through physical damage of the endothelial layer of the blood vessel, causes the exposure of TF to circulating blood, making it accessible to circulating factor VII (FVII) [13-16]. This coagulation factor binds to TF with very high affinity and specificity [17]. Once bound to TF, FVII is rapidly converted to its activated form (FVIIa) *via* limited proteolysis [18-19] (Fig. **2**). Activated FVII binds Factor X (FX), that, in turn, is converted in its activated form (FXa). Then, the extrinsic pathway continues, leading to thrombin activation and clot formation [19]. Many coagulation proteases such as factors IXa, Xa, XIIa, thrombin and plasmin [18-24] are able to amplify this activation process since they cause the direct activation of FVII to FVIIa. More important, the TF:VIIa complex can itself catalyze the activation of FVII bound to TF, *via* an auto activation reaction [25-26], in which is involved FXa too. Although the FVII in plasma circulates as a zymogen, it has been demonstrated that normal individuals might have low levels of activated factor VII (FVIIa) in their plasma (about 1% or less of the total factor VII) with an unknown role [27].

FVIIa is an extremely weak serine protease on its own, but its enzymatic activity is enhanced dramatically when it binds to TF [17]. Specifically, TF/FVIIa binding significantly increases FVIIa ability to catalyze the hydrolysis of small peptidyl amide and ester substrates from 20- to 100-fold, and this phenomenon is closely dependent upon the substrate [28, 29]. Substrate hydrolysis by serine proteases is known to be a multi-step process, and any of the steps along the reaction pathway might be affected in the allosteric activation of FVIIa by TF. Because TF is an integral membrane protein, the TF/VIIa complex is always tethered to the membrane surface. This has two important consequences: first, the coagulation cascade is activated only where it is needed, i.e. at sites of vascular injury; second, binding of FVIIa to TF activates a number of intracellular signals that culminate in cell proliferation and new gene expression, including inflammatory genes [30-32].

It has been described that procoagulant activity of intact cells that express TF on their surface is significantly lower if compared with the activity measurable in the same cells when damaged, lysed, or treated with calcium ionophore [33]. Indeed, although TF is present on the surface of such cells, it becomes fully active only when the membrane properties of the cell are altered [34, 35]. In particular, it has been described a phenomenon called “TF encryption”. It is known that the distribution of aminophospholipids (such as the negatively charged phosphatidylserine) is restricted to the inner leaflet of the plasma membrane of the cells. Negatively charged phospholipids are required for substrate molecules such as factors IX or X to bind to the membrane, so their sequestration limits the activity of TF on cell surface. When cells are lysed, damaged or treated with calcium ionophore, this phospholipid asymmetry is lost. Moreover, in some cell types, TF may associate with caveolae, which are areas of the cell surface with altered lipid composition. Again, it has been proposed that dimerization or oligomerization of TF in the membrane may reduce its activity, and that damage or lysis of cells may promote the formation of active TF monomers.

Pathophysiology of coagulation is tightly regulated by another important protein known as Tissue Factor Pathway Inhibitor (TFPI), that is the endogenous inhibitor of the extrinsic coagulation pathway; specifically, TFPI is a potent inhibitor of the TF/FVIIa complex, and its action is related to the presence of FXa [36] (Fig. **2**). TFPI is composed of three Kunitz-type protease inhibitor domains: the first Kunitz domain reacts with the active site of FVIIa in the TF:VIIa complex [37], while the second Kunitz domain reacts with the active site of FXa. Once the TFPI:Xa complex forms, it binds with higher affinity to TF:VIIa than does the TFPI molecule alone; this results in the formation of a fully inhibited tetramolecular complex TF:VlIa:TFPI:Xa [37, 38]. Much of the circulating TFPI is bound to lipoproteins [39-40]; this form represents about 50% to 60% of the total circulating TFPI, whereas carrier-free TFPI represents about 20% of the total. A third pool of TFPI is confined to platelets, which carry approximately 10% of the total TFPI [41]. The *in vivo* infusion of heparin increases the circulating levels of TFPI in plasma 2- to 4-fold [42, 43]. The source of this additional TFPI is thought to be the endothelium, at the surface of which TFPI is bound. The TFPI released by heparin *in vivo* represents the carrier-free molecule, which might be biologically most active [38]. TFPI also promotes the internalization and degradation of TF:VIIa complexes on the surface of monocytes [44], thus subtracting these complexes to coagulation cascade.

### TF/FVIIa in Cardiovascular Disease

Several experimental and clinical studies have clearly demonstrated that the complex TF/FVIIa is the key initiator of the coagulation cascade in cardiovascular disease [1].

TF expression represents the link explaining the relationship existing among several chemical mediators and the pathophysiology of coronary artery disease as well as of its main complication represented by acute coronary syndromes. In fact, it has been demonstrated that these mediators might exert their effects by inducing TF expression. Specifically, inflammatory markers such as C-Reactive Protein or neopterin as well as smoke-derivative substances might play an important role in acute coronary syndromes since it has been demonstrated that they are active partaker in triggering coronary TF-mediated coagulation [15, 45, 46]. In addition, molecules involved in pathophysiology of other cardiovascular co-morbidity such as urotensin II or angiotensin have been associated with TF expression to explain their mechanism of action [13, 47].

Moreover, oxygen free radicals, endogenously generated upon post-ischemic reperfusion, induce TF-mRNA transcription and expression of TF procoagulant activity. As seen in in ex vivo and *in vivo* hearts subjected to ischemia and reperfusion, a condition associated with a production of oxygen free radicals in large amounts, a marked increase in TF activity occurred. This increase was accompanied by a significant impairment of coronary flow during reperfusion and possibly contribute to the occurrence of reperfusion injury [9].

Immunohistochemistry studies have demonstrated that TF can be detected in several cell types, such as monocytes, foam cells, and fibroblasts isolated from human atherosclerotic coronary and carotid plaques [3, 48]. Interestingly, TF of human atherosclerotic plaques retains its full procoagulant properties [1]. Moreover, in patients with clinical evidence of acute coronary syndromes, TF antigen levels and TF procoagulant activity measured in human atherectomy specimens were significantly higher that those measurable in specimens obtained from patients with stable angina [49]. Conversely, TF was rarely detected in patients with restenosis lesions even if the resulting clinical presentation was an unstable coronary syndrome. Tissue Factor was readily detected in de novo lesions in patients with unstable coronary syndromes, suggesting a role for TF in the pathogenesis of this disease process [4]. Again, Randi *et al*, analyzing gene expression in coronary plaques from patients with stable or unstable angina using gene arrays, demonstrated higher TF expression in unstable angina samples [50]. Furthermore, plasma TF activity seems to have impact on prognosis in patients with ACS. Steppich *et al* demonstrated that systemic TF activity in acute myocardial infarction has an unfavorable prognostic value and, as a marker for altered coagulation, it might predict the atherothrombotic risk [51].

Plasma levels of FVIIa seem to be an another key point of the coagulation cascade. Bozzini *et al*. showed that polymorphisms in the factor VII gene promoter on activated factor VII levels may modulate the risk of myocardial infarction in males with advanced coronary artery disease [52]. In addition, a recent study has demonstrated that C-reactive protein plasma levels were related with FVII concentration in patients with coronary artery disease [53]. Moreover, activity of FVII may be considered as an independent cardiovascular risk factor. Specifically, Karatela *et al*. have recently demonstrated that raised FVII and leptin levels in coronary artery disease (CHD) patients were independently associated with insulin resistance; this was not observed among the non-CHD subjects [54].

Taken together, these data clearly underline the importance of TF:FVIIa as one of the main determinant of human atherosclerotic plaque thrombogenicity.

### TF/VIIa Complex Inhibitors in Cardiovascular Disease

#### Tissue Factor Pathway Inhibitor

Since TFPI has an important role in modulating the activity of the TF:FVIIa complex (Fig. **2**), recombinant human TFPI might be useful in patients with acute coronary syndromes. Thus, this protein has been successfully expressed in a variety of hosts, including bacteria, and has been shown to be effective in preventing thrombus formation in a variety of experimental models. Haskel *et al*. [55] for the first time demonstrated that administration of pharmacological doses of human recombinant TFPI was associated with lack of reocclusion after discontinuation of t-PA in a canine model of coronary thrombolysis. Other experimental studies have also shown that recombinant TFPI was effective in inhibiting intravascular thrombosis, but this effect was achieved at doses far higher than those physiologically measurable in plasma [56]. Not surprisingly, therefore, considering also the FXa inhibitory effects of TFPI, Oltrona *et al*. [57] found that systemic administration of recombinant TFPI led to a marked prolongation in PT, suggesting that recombinant TFPI at doses effective in preventing arterial thrombosis might be associated with a substantial risk of bleeding. St. Pierre *et al*. [58] demonstrated that recombinant TFPI administration after balloon overstretch insult to the carotid arteries in pigs reduced TF expression, FXa activity, and attenuated accumulation of thrombus at the site of insult. Finally, in a clinical trial in patients with sepsis, recombinant TFPI showed promise in reducing mortality in critically ill sepsis patients [59]. However, several clinical trials aimed to evaluate TFPI systemic administration in patients with acute coronary syndromes have been interrupted for ethical reasons since the risk of bleeding increased significantly. Fortunately, the rapid progress of *in vivo* gene transfer technologies has created powerful new tools to transfer foreign genes into the cells of a variety of organs, including the vascular wall. Different vectors have been developed to efficiently transfect target cells, including retroviral, adenoviral, and direct DNA transfer. Therefore, giving the feasibility of transfecting the arterial wall with foreign genes, it is of no surprise that TFPI has been the focus of several studies in this field. The main theoretical advantage of increasing local TFPI concentrations by gene transfer to the arterial wall is that therapeutic TFPI levels can be achieved only where they are needed, i.e., at the damaged arterial site where TF is exposed, without concomitant, potentially dangerous systemic effects. Thus, starting from these considerations, several studies have clearly demonstrated the antithrombotic efficacy of arterial TFPI gene transfer in different models of intravascular thrombosis [60, 61]. However, to date, considering ethical concerns regarding “gene therapy” in humans, this kind of pharmacological approach to inhibit TF/FVIIa complex seems to be far.

#### rFVIIai

**Fig. (3):**
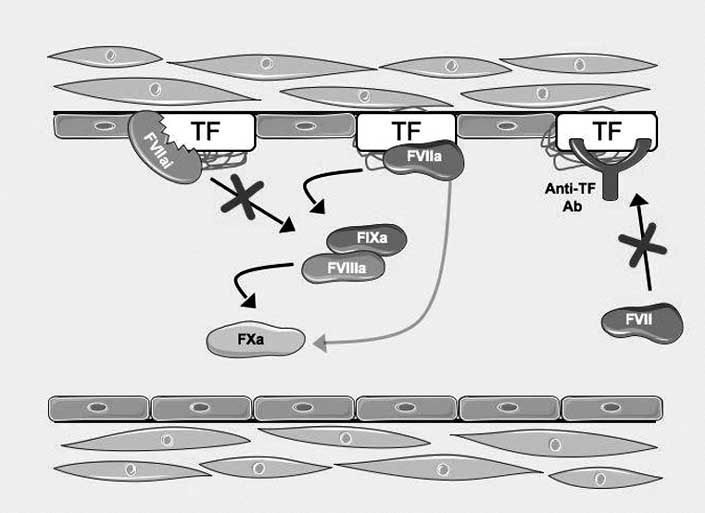
Examples of TF/VIIa complex inhibitors. Anti-TF monoclonal antibody binds TF and prevent FVII binding. rFVIIai is enzymatically inactive but it has high TF binding affinity. Thus, it exerts its antithrombotic effect by competing with FVIIa for TF binding and consequently impeding TF/FVIIa activity.

Recently, human recombinant FVIIa in which the active site of FVIIa is blocked with a covalent inhibitor, such as chloromethylketone (known as rFVIIai), has been produced and successfully used to block TF:VIIa procoagulant activity. rFVIIai retains its TF binding capacity but is enzymatically inactive. This molecule exerts its antithrombotic effect by competing with native factor VIIa (FVIIa) for TF binding. Since it has a significantly higher affinity to TF than native FVIIa, it avoids that coagulation cascade could proceed downstream [62-64] (Fig. **3**).

Immunohistochemical studies have evidenced that rFVIIAi can be detected at site of arterial injury in vessel sections obtained from animals 24 h following rFVIIai administration, despite at this time the compound was completely eliminated from the circulation. In different animal models, rFVIIai efficiently prevented TF-induced arterial thrombosis, without any concomitant potentially hazardous systemic effects [65-67]. Interestingly, and in line with histological observations, rFVIIai-dependent inhibition of arterial thrombosis could be observed despite plasma concentrations were undetectable, thus witnessing that this molecule exerted its effect only at site of injury [68].

The potential role of rFVIIai in cardiovascular disease has been demonstrated in ischemia/reperfusion too [69]. Recently, it has been demonstrated that this rFVIIai effect is primarily due to rFVIIai ability to reduce inflammation-related lethal I/R injury. Inhibition of toll-like receptor-4 (TLR-4) and of nuclear factor-kappaB (NF-kB) mediated cell signalling might be involved. Specifically, levels of NF-kB and of NF-kB-dependent protein such as TF and IL-6, usually increased after ischemia/reperfusion were significantly reduced after rFVIIai administration [70].

A phase I clinical study has reported that administration of single doses of rFVIIa up to 400 mcg/kg to 64 healthy subjects did not affect the safety of the subjects nor the hemostatic function, except for the expected prolongation of the prothrombin time (PT) [71]. On the basis of these findings, a multicenter, double-blind, dose-escalation, randomized trial evaluating the efficacy and safety of rFVIIa in patients undergoing elective or urgent PCI was performed [72], and in association with this trial, a substudy was designed to evaluate the antithrombotic effect of FFR-rFVIIa in an ex vivo perfusion flow chamber connected directly to the patients’ blood streams [73]. These studies demonstrated that FFR-rFVIIa has a potent antithrombotic effect at different shear rates and severe arterial injury conditions, suggesting a potential use for this molecule in this clinical setting.

#### Other TF/FVIIa Inhibitors

Another potential option to interfere with TF/FVIIa complex is represented by antibodies directed against TF: binding of these antibodies prevents FVII interaction with its natural ligand. One of the first antibody able to interfere with TF/ FVIIa complex was AP-1, a monoclonal antibody raised against rabbit TF. AP-1 has been proven to block TF-procoa-gulant activity *in vitro* and *in vivo* at very low concentrations [2] (Fig. **3**). In particular, administration of AP-1 to rabbits with recurrent thrombosis of the carotid artery was associated with a complete inhibition of thrombosis without a concomitant prolongation in systemic hemostatic parameters or an alteration in platelet aggregation [2]. The same agent has also been shown to accelerate the thrombolytic properties of t-PA and prevent reocclusion after its discontinuation in a rabbit model of carotid artery thrombosis and thrombolysis [5].

Recently, it has been developed a chimeric mousehuman monoclonal antibody directed against TF and known as ALT836. This antibody binds to TF at its FX binding site. In patients with stable coronary artery disease enrolled in the PROXIMATE-TIMI 27 trial, this antibody had an interesting dose-dependent anticoagulant effect without any significant side effect such as bleeding [74].

Another potent TF:VIIa complex inhibitor is known as XK1. It is a chimeric protein which consists of the light chain of FXa linked to the first Kunitz domain of TFPI [75]. Other hybrid proteins with increased affinity for TF/FVIIa complex have also been genetically engineered to inhibit activation of coagulation cascade [76, 77], and a version with even greater potency for FVIIa has been created by linking a modified Kunitz-type inhibitor with a mutated form of soluble TF [78]. In addition, NAPc2, an inhibitor of the TF/VIIa complex, has been cloned from hookworms [79]. This molecule exerts its effects by binding to FXa and has an inhibitory mechanism resembling that of TFPI. The antithrombotic effect of NAPc2 has recently been demonstrated in a dose-finding study on the prevention of venous thromboembolism in patients undergoing total knee replacement [80].

#### Future Directions

As reported above, TF/FVIIa inhibitors have been successfully tested *in vivo* after parenteral administration, but research of an orally bio-available drug still remains an undiscovered field. Comforting results observed in preclinical models and clinical trials [81, 82] in which small protein and antibody-based inhibitors of the TF/FVIIa pathway have been tested, have stimulated several future studies aimed to develop orally active TF/FVIIa inhibitors and to perform a tailored anti-thrombotic therapy. Probably, the main limitation that should be overcome is that an effective oral drug will require a careful balance between optimal inhibitor characteristics and drug-like or pharmacokinetic properties, that is a challenge which often does not have an easy solution.

Moreover, since TF/FVIIa inhibitors might be useful also in preventing other TF-mediated phenomena, such as inflammation and cell proliferation, these molecules should be tailored to exert their effects only where they are needed, without affecting the physiological haemostasis.

In interventional cardiology area, particularly attractive could be the engineering of a balloon coated with an inhibitor of TF/FVIIa, such as rFVIIai able to permit local drug release at site of injury/thrombosis, during percutaneous transluminal coronary angioplasty (PTCA). Alternatively, a TFPI-coated balloon might be used in the same clinical setting, in order to increase local TFPI concentrations and obtain a local antithrombotic effect. Finally, a fascinating hypothesis might be that of a balloon coated with genes codifying for TFPI, to directly obtain gene transfer to the arterial wall, in order to facilitate the stabilization of active atherosclerotic plaque before stent deployment.

## CONCLUSIONS

TF:VIIa complex represents the “critical point” of the extrinsic pathway of blood coagulation. Thus, it is intuitive why it has become an attractive tool for the development of newer antithrombotic agents able to prevent complex formation or to inhibit its catalytic activity. This kind of antithrombotic therapy has several theoretical advantages if compared with other interventions directed against other “downstream” components of the coagulation cascade, such as heparin and its derivatives or direct antithrombin agents.

Indeed, although potent synthetic inhibitors of TF/FVIIa had been discovered and tested in animal models, any of these have advanced into clinical trials. The systemic effects observed and, specifically, the marked elongation of bleeding time observed in experimental studies have highlighted that safety, effective dose and route of administration are the main issues to resolve. On the other side, while the local delivery of genes or drugs during interventional procedures seems very intriguing, the feasibility of this approach needs to be demonstrated.

However, in spite of these considerations, TF:FVIIa complex still remains a challenging and important target to study and develop future generations of antithrombotic agents.

## Figures and Tables

**Table 1:** TF/VIIa Complex Inhibitors: Mechanism of Action, Effects on Bleeding and Trials in Cardiovascular Disease

	MECHANISM	RISK OF BLEEDING	TRIAL IN CARDIOVASCULAR DISEASE
rTFPI	Inhibition of TF/FVIIa complex *via* FXa/rTFPI complex	↑	NO
rFVIIai	Competition with native FVIIa for TF binding	↔	YES
ALT-836	Chimeric antibody against TF	↔	YES
XK1	TFPI-like mechanism	↔	NO
NAPc2	TFPI-like mechanism	↔	YES

## ABBREVIATIONS

TF: = Tissue factor
LPS: = Lypopolysaccharide
FVIIa: = Factor VII activated
FXa: = Factor X activated
FIXa: = Factor IX activated
FXIIa: = Factor XII avtivated
TFPI: = Tissue factor pathway inhibitor
CHD: = Coronary artery disease
AP-1: = Monoclonal antibody raised against rabbit TF
rFVIIai: = Recombinant FVIIa with active site blocked with a covalent inhibitor
PTCA: = Percutaneous transluminal coronary angioplasty
